# A Model for the Determination of Potato Tuber Mass by the Measurement of Carbon Dioxide Concentration

**DOI:** 10.3390/plants12162962

**Published:** 2023-08-16

**Authors:** Boris Rumiantsev, Sofya Dzhatdoeva, Elchin Sadykhov, Azret Kochkarov

**Affiliations:** Federal Research Centre “Fundamentals of Biotechnology” of the Russian Academy of Sciences, Leninsky Prospect, 33, Build. 2, 119071 Moscow, Russia

**Keywords:** precision agriculture, vegetation, diagnostics, potato, tuber, yield, carbon dioxide, photosynthesis, vertical farm

## Abstract

The implementation of advanced precision farming systems, which are becoming relevant due to rapid technological development, requires the invention of new approaches to the diagnostics and control of the growing process of cultivated crops. This is especially relevant for potato, as it is one of the most demanded crops in the world. In the present work, an analytic model of the dependence of potato tubers mass on carbon dioxide concentration under cultivation in a closed vegetation system is presented. The model is based on the quantitative description of starch molecule synthesis from carbon dioxide under photosynthesis. In the frame of this work, a comprehensive description of the proposed model is presented, and the verification of this model was conducted on the basis of experimental data from a closed urban vertical farm with automated climate control. The described model can serve as a basis for the non-contact non-invasive real-time measurement of potato tuber mass under growth in closed vegetation systems, such as vertical farms and greenhouses, as well as orbital and space crop production systems.

## 1. Introduction

Currently, potatoes are one of the most popular agricultural crops in the world, along with corn, wheat, and sugar beet [1]. This is due to its relative unpretentiousness [2] and the corresponding wide range of possible climatic growing conditions [3], relatively fast vegetation rate (about 80–100 days are required for potato ripening), and high yields (at the level of 15–20 t/ha [4]). Due to the wide geographical distribution of potatoes, the methods of its cultivation are also diverse—for example, potato is grown in fields [5], in greenhouses [6], and vertical farms [7], as well as using aeroponics methods [8]. Moreover, the possibility of growing potatoes in the conditions of spacecraft [9] and on neighboring celestial bodies as a part of their exploration is being discussed [10,11]. The possibility of such methods of cultivation is indicated by studies conducted to date on the cultivation of crops in space conditions on the International Space Station [12], during which it was shown that crops such as lettuce and cabbage can successfully grow under conditions of low gravity and increased radiation levels.

Potato cultivation in closed urban vertical farms under controlled conditions is becoming especially relevant at the present time against the background of climate change, growth of the human population [13], and global urbanization [14]. Due to these factors, even though at the present time growing in vertical farms is cost-effective only in the case of seed potatoes [7], growing potatoes for consumption in such a way may become necessary in the foreseeable future. Climate change is characterized by a long-term shift in global weather patterns, including changes in temperature, precipitation, and wind patterns. Climate change also leads to more frequent extreme weather events like heatwaves, droughts, floods, and storms, which can have significant impacts on agriculture and food supply systems [15]. Overcoming these difficulties implies the creation of sustainable agricultural systems, where growing conditions do not depend on environmental factors. At the same time, human population growth forces the development of new approaches to cultivation, allowing one to get a guaranteed high yield throughout the year. Finally, global urbanization, characterized by the increase in the urban population percentage, requires the development of automated solutions for cultivation. These requirements, dictated by climate change, population growth and global urbanization, can be met by introducing closed urban vertical farms with automatically controlled cultivation conditions.

The implementation of projects of this kind requires an extensive set of methods and technologies aimed at diagnostics and controlling the process of plant vegetation [16]. Of particular interest in this direction is the possibility of non-contact measurement of the mass of formed tubers in a real-time regime. The scientific relevance of such measurements is due to the possibility of testing the impact of external factors (such as temperature, lighting and irrigation mode, carbon dioxide concentration) on the process of plant vegetation and tuber formation. At the same time, from an applied point of view, the relevance of such measurements is due to the possibility of predicting potato yields directly at the vegetation stage.

Direct implementation of such measurements is difficult due to the requirement of non-invasiveness, which limits the range of possible technologies to those based on the registration of electromagnetic and/or acoustic waves with the use of tomography approaches [17]. However, such measurements can be carried out indirectly on the basis of measuring the dynamics of parameters related to the mass of the formed tubers and the corresponding recalculation of the values of these parameters into the mass of tubers based on the calculation model.

To date, a number of software packages have been developed [18], aimed at modeling the processes of potato vegetation, which allow calculating the mass of tubers in accordance with the specified growing conditions. Thus, in particular, the SUBSTOR-POTATO software package has made it possible to simulate the potato yield under conditions of high nitrogen content in the soil [19], the LINTUL-Potato program code has been used to predict the yield and size of potato tubers [20], and the WOFOST (World Food Studies) model has been used to determine the effect of different climate change scenarios on potato productivity [21]. Another model, that can be used for different crop types, is the QUEFTS (QUantitative Evaluation of the Fertility of Tropical Soils) model [22], that can be used for the estimation of crop yield by given soil properties and the amount of applied fertilizer. This model has been used for potato [23], as well as for other crops [24,25]. In addition, considering other crops, it is worth noting that today there are also a number of models for the simulation of fruit tree development [26].

The availability of such software packages makes it possible to carry out numerical experiments to determine the yield of potatoes when grown under given conditions.

Such software models generalize empirical statistical information about the characteristics of different potato varieties under different environmental conditions to make forecasts of their response to a new combination of external factors. As a rule, they are used to model processes over a large area—on the scale of a field, a region, and even on a global scale. However, these models have limitations when modeling the potato growing process on a small scale, such as, for example, a vertical farm, greenhouse or a single plant, which limits the use of these models in the area of precision agriculture [27,28]. The inability to scale such models towards small scales is partly due to the statistical nature of the data used in the models. In addition, such universal models may be impractical in experimental applications if they require raw data that is not easy to measure. Thus, the complexity of such comprehensive models and the difficulty of scaling make them difficult to apply in the framework of experiments.

As a result, the direct integration of such models into a real-time diagnostic system seems difficult to implement. In this regard, within the framework of this work, a clear analytical calculation model has been developed and verified, which allows calculating the dynamics of the potato tuber mass according to given dynamics of the concentration of carbon dioxide in the growing room. The analytical nature of the calculations within the framework of the model indicates the possibility of embedding this model into a real system for diagnostics of the tuber formation process, operating in real time and not requiring serious computational power. Thus, the purpose of this work is to develop and verify an analytical model linking the dynamics of the mass of potato tubers grown in a closed room and the concentration of carbon dioxide. Within the framework of this goal, the tasks were, firstly, to make the quantitative description of the relationship between the mass of potato tubers and the concentration of carbon dioxide in the cultivation room on the basis of a chemical description of the process of converting carbon dioxide molecules into starch molecules in the framework of photosynthesis and, secondly, to test the developed model with the use of experimental and literary data by the comparison of the calculated tuber yield with the experimental one.

## 2. Results and Discussion

The absorption of carbon dioxide (CO_2_) by a potato plant is a necessary condition for its vital activity, since CO_2_ is one of the components involved in the metabolism and formation of plant biomass, which makes it possible to use the rate and absorption of CO_2_ and its integral amount as a marker of the process of formation of plant biomass.

The formation of plant biomass, which, in the case of potatoes, includes tubers, occurs during photosynthesis. Therefore, the process of plant biomass formation and, consequently, the rate and amount of absorbed CO_2_ are influenced by environmental factors that determine the rate of photosynthesis. Such factors include the intensity of light and its spectral composition, as well as the ambient temperature, air, and soil humidity.

Measuring the dynamics of the rate of CO_2_ absorption by a plant allows both determination of the dynamics of its biomass and tracking of the influence of external factors—lighting conditions and microclimate parameters—on the plant’s vital activity. Below, the model connecting the dynamics of CO_2_ concentration and the mass of potato tubers in the framework of its cultivation in a sealed cultivation room is presented.

It is worth noting that the influence of environmental factors such as temperature, light intensity, air, and soil humidity is taken into account in the proposed model indirectly, since the input data for the model comprise the dynamics of CO_2_ determined by the absorption rate of this gas by the plant. Accordingly, all environmental factors that will affect the rate of photosynthesis and, consequently, the rate of CO_2_ absorption will be indirectly taken into account in the model. Thus, there is no direct consideration of environmental factors in the form of input parameters in the model, and all influence of the environmental parameters is contained in the dynamics of CO_2_ concentration, which is the input parameter of the model.

In the frame of the proposed model, the term “sealed cultivation room” means a room that has no exchange of CO_2_ with the external environment and, as a consequence, no exchange of air. The initial amount of CO_2_ pumped up to the sealed cultivation room is absorbed by the plant under its vegetation process. In this type of room, the CO_2_ concentration dynamics are determined completely by the process of CO_2_ absorption and release by the plant, which allows direct linking of the measured CO_2_ concentration dynamics with the photosynthesis process and, in the case of potato, with the mass of the growing tubers.

Other environmental conditions in the sealed cultivation room which influence rate of photosynthesis and, consequently, the dynamics of CO_2_ concentration in the room, such as temperature, lighting conditions, and humidity of air and soil, can be arbitrary. Dynamic change of these conditions under the cultivation process leads to the change in the CO_2_ concentration dynamics in the sealed room. This is directly taken into account in the proposed model, where, as mentioned above, CO_2_ concentration serves as an input parameter.

Extension of the model for the case of an unsealed room can be realized in two ways. The first way is to consider the case of elevated CO_2_ partial pressure in the room, which, together with the other air constituents, will lead to the excess of resulting pressure in the room over the air pressure in the external environment. In this case, there will be continuous CO_2_ leakage from the cultivation room to the external environment, that can be easily taken into account in the model. The second way is to consider the case of controlled exchange of the CO_2_ with the external environment, when the amount of CO_2_ is pumped up to the room at given moments during vegetation process, that also can be introduce to the proposed model.

Below, the proposed model is discussed in its simple form, i.e., in the case of the sealed room. Nevertheless, even such a simple formulation makes it possible to apply the model within the framework of real tasks, such as growing in closed automated containers, which may be relevant in space and Arctic missions.

### Description of the Proposed Model

One of the main chemical compounds that make up potato tubers is starch, the chemical formula of the monomer of which is C_6_H_10_O_5_. Depending on the potato variety, the mass fraction of starch in tubers is $\eta_{starch}$ = 10–25% [29]. Based on this, knowing the mass of all starch monomer molecules $m_{starch}\left(t\right)$ stored in the tubers of the plant at time t, it is possible to determine the total mass of tubers $m_{tubers}\left(t\right)$ at the moment t in accordance with the equation

(1) $$m_{tubers}\left(t\right)=\frac{m_{starch}\left(t\right)}{\eta_{starch}}$$

The mass of all starch monomer molecules $m_{starch}\left(t\right)$ stored in the tubers of the plant can be expressed as

(2) $$m_{starch}\left(t\right)=m_{1}\cdot N_{starch}\left(t\right)$$

where $m_{1}\approx 27\cdot 10^{-26}\;\mathrm{kg}$ is the mass of starch monomer molecule C_6_H_10_O_5_, $N_{starch}\left(t\right)$ is a number of starch monomer molecules C_6_H_10_O_5_, stored in the tubers of the plant by the moment of time t.

**Figure 1:**
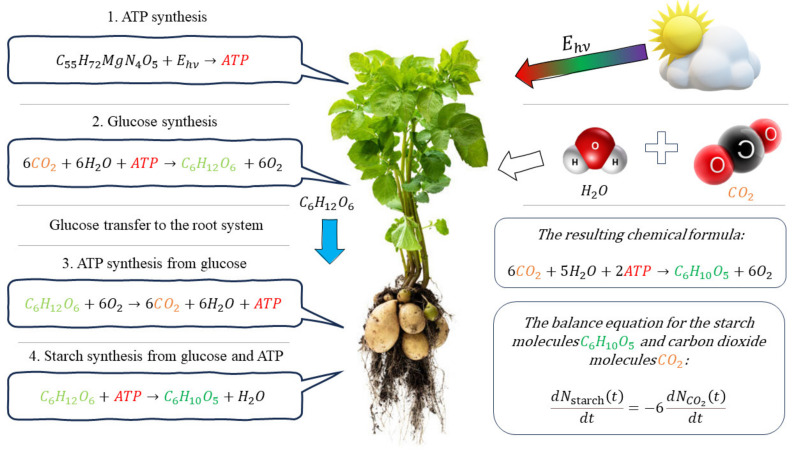
Scheme of starch synthesis in potato. The red color in the chemical formulae corresponds to adenosine triphosphate (ATP), the orange corresponds to carbon dioxide (CO_2_), the light green color corresponds to glucose (C_6_H_12_O_6_), the dark green color corresponds to the starch monomer (C_6_H_10_O_5_).

To determine the relationship between the number of starch monomer molecules and the number of CO_2_ molecules used in the synthesis of the former, it is necessary to consider the process of photosynthesis. Starch is synthesized by the plant as a result of the process of photosynthesis, which, in the case of potatoes, can be described in the form of four consecutive stages [30,31] (Figure 1).

At the first stage, a molecule of adenosine triphosphate (ATP) is synthesized, in which the energy of light falling on the plant leaves containing chlorophyll is stored. This stage can be expressed by the chemical formula

(3) $$C_{55}H_{72}{\mathrm{MgN}}_{4}O_{5}+E_{hv}\to \mathrm{ATP}$$

where C_55_H_72_MgN_4_O_5_ is a chlorophyll molecule, E_hv_ is the energy of light radiation, ATP is an ATP molecule. At the second stage, the monomer of the glucose molecule (C_6_H_12_O_6_) is synthesized due to the reaction between a carbon dioxide molecule (CO_2_) absorbed from the environment and a water molecule (H_2_O) in the presence of an ATP molecule providing energy for this reaction [32]:

(4) $$6{\mathrm{CO}}_{2}+6H_{2}O+\mathrm{ATP}\to C_{6}H_{12}O_{6}+6O_{2}$$

This reaction, as well as the reaction of ATP synthesis (3), takes place in the aerial part of the plant. Since the starch molecule undergoes hydrolysis and, as a result, cannot be transferred in the form of an aqueous solution to the tuber, its synthesis occurs directly in the tuber. For this, the synthesized glucose molecule C_6_H_12_O_6_ in the form of an aqueous solution is transferred to the tuber, where one of two processes occurs with its participation: either the release of energy in the form of an ATP molecule, or the synthesis of a starch monomer molecule (C_6_H_10_O_5_) using the energy stored in ATP, which is obtained as a result of the breakdown of the previous glucose molecule C_6_H_12_O_6_. The first of these processes can be written as

(5) $$C_{6}H_{12}O_{6}+6O_{2}\to 6{\mathrm{CO}}_{2}+6H_{2}O+\mathrm{ATP}$$

The second process can be described as:

(6) $$C_{6}H_{12}O_{6}+\mathrm{ATP}\to C_{6}H_{10}O_{5}+H_{2}O$$

By substituting the ATP value obtained from (5) into Equation (6), as well as substituting the C_6_H_12_O_6_ value expressed from (4), it is possible to obtain the resulting balanced equation describing the mentioned stages of the synthesis of the starch monomer molecule C_6_H_10_O_5_:

(7) $$6{\mathrm{CO}}_{2}+5H_{2}O+2\mathrm{ATP}\to C_{6}H_{10}O_{5}+6O_{2}$$

Thus, as evidenced by Equation (7), the synthesis of one molecule of starch monomer C_6_H_10_O_5_ in a plant occurs with the participation of six molecules of carbon dioxide CO_2_.

In accordance with (7), the rate of change in the number of starch monomer molecules C_6_H_10_O_5_ stored in plant tubers can be written as

(8) $$\frac{dN_{starch}\left(t\right)}{dt}=-6\frac{dN_{CO_{2}}\left(t\right)}{dt}$$

where $N_{CO_{2}}\left(t\right)$ is the number of carbon dioxide molecules CO_2_ at time t. The solution of the differential Equation (8) has the form

(9) $$N_{starch}\left(t\right)=N_{starch}\left(0\right)-6\left(N_{CO_{2}}\left(t\right)-N_{CO_{2}}\left(0\right)\right)$$

where $N_{starch}\left(0\right)$ is the number of starch monomer molecules C_6_H_10_O_5_ in plant tubers at time t=0 (when planting), $N_{CO_{2}}\left(0\right)$ is the number of carbon dioxide molecules CO_2_ at time t=0 (when planting). Taking into account (9) and (2), the equation for the mass of plant tubers can be rewritten as

(10) $$m_{кл}\left(t\right)=\frac{m_{1}}{\eta_{кp}}\cdot \left(N_{кp}\left(0\right)-6\left(N_{CO_{2}}\left(t\right)-N_{CO_{2}}\left(0\right)\right)\right)$$

Equation (10) allows using the measured values of the number of starch monomer molecules C_6_H_10_O_5_ and carbon dioxide CO_2_ to determine the mass of plant tubers. Since direct measurement of the number of molecules seems difficult to implement from a practical point of view, it is reasonable to switch to experimentally measured values in Equation (10). The number of starch monomer molecules $N_{кp}\left(0\right)$ can be expressed as

(11) $$N_{starch}\left(0\right)=\frac{m_{starch}\left(0\right)}{m_{1}}=\frac{\eta_{starch}\cdot m_{tubers}\left(0\right)}{m_{1}}$$

where $m_{tubers}\left(0\right)$ is the mass of the tuber at planting. In turn, the number of carbon dioxide molecules $N_{CO_{2}}\left(t\right)$ can be expressed as:

(12) $$N_{CO_{2}}\left(t\right)=n_{CO_{2}}\left(t\right)\cdot V$$

where $n_{CO_{2}}\left(t\right)$ is the concentration of carbon dioxide molecules in the room at time t, and V is the volume of the room. Taking into account (12) and (11), Equation (10) can be written as:

(13) $$m_{tubers}\left(t\right)=m_{tubers}\left(0\right)+6\frac{m_{1}}{\eta_{starch}}V\cdot \left(n_{CO_{2}}\left(0\right)-n_{CO_{2}}\left(t\right)\right)$$

Thus, the Equation (13) allows determination of the tuber mass $m_{tubers}\left(t\right)$ formed by the time t by measuring the concentration of carbon dioxide in the room $n_{CO_{2}}\left(t\right)$ at the moment t. In order to do this, it is necessary to know the mass of the tuber $m_{tubers}\left(0\right)$ and the concentration of carbon dioxide in the room $n_{CO_{2}}\left(0\right)$ at the time of planting (t=0), as well as the volume of the room V and the variety-specific mass fraction of starch in the tubers $\eta_{starch}$. It is important to note that the considered model is valid for a sealed room in which there is no exchange of carbon dioxide with the environment. Accounting for additional controlled supply and removal of carbon dioxide into a cultivation room, when the intensity and duration of gas exchange are known, can be made by modifying the Equation (8).

The current model contributes to the field of research on the CO_2_ absorption by plants and its conversion into plant biomass during photosynthesis. In the paper [33], the researchers focus on the refinement of the CO_2_ absorption model by determination of the influence of temperature, vapor pressure deficit, and elevation on the CO_2_ absorption rate for use in large-scale Earth System Models. However, the paper does not discuss the process of CO_2_ conversion to biomass in detail. The paper [34] is focused on the study of the dependence of potato plant biomass on temperature based on the dependence of the photosynthesis rate on temperature, where CO_2_ absorption rate is used as a photosynthesis marker. This work includes a model for calculating the amount of CO_2_ absorbed by potatoes during cultivation in an unsealed container under elevated levels of CO_2_, which allowed taking into account CO_2_ leakage rate in the model. Additionally, it discusses an experiment aimed to establish a relationship between the amount of absorbed CO_2_ and the mass of tubers, in which it was shown that the tuber mass is linearly proportional to the mass of absorbed CO_2_, which is in agreement with the proposed model (see Equation (13)). However, in the frame of this work [34], there is no detailed description of the established relationship at the molecular level.

Compared to these works, the developed model in the present paper is focused on the quantitative description of the CO_2_ conversion to the potato tuber mass based on the first principles at the molecular level for the case of potato cultivation in the sealed room, i.e., where is no CO_2_ exchange with the external environment. It should be noted that in the frame of the approach used in [34], when cultivation occurred in the unsealed room under elevated levels of CO_2_, it is possible to modify the proposed calculation model by taking into account the leakage rate and use it in the case of cultivation in an unsealed room, as was mentioned above.

## 3. Materials and Methods

### Verification of the Proposed Model

Measuring the dynamics of CO_2_ concentration in an unsealed room, in which there is an uncontrolled exchange of CO_2_ with the environment, does not allow direct verification of the model based on comparison of the experimental dependence $m_{tubers}\left(n_{CO_{2}}\right)$ with the calculated dependence determined by Equation (13). Despite this, it is possible to verify the described model by comparing the calculation results based on literature and experimental data for potatoes grown in the urban vertical farm of the Federal Research Center “Fundamentals of Biotechnology” of the Russian Academy of Sciences with automated climate control, the growing conditions in which are described in detail in [7].

**Figure 2:**
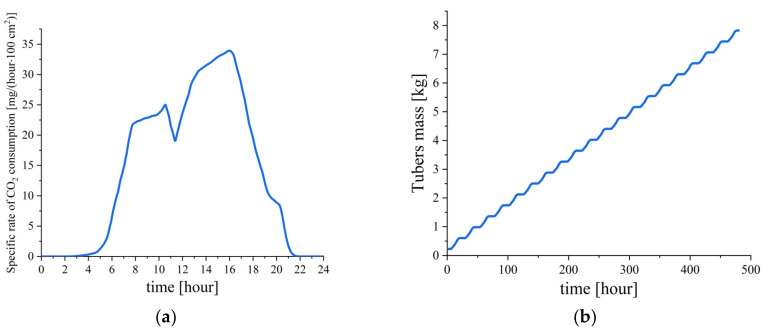
(**a**) The dependence of the specific (per unit area of leaves) rate of CO_2_ absorption by potato leaves according to [35]; (**b**) the calculated dependence of the tuber mass on the scale of the tuber formation period, calculated from experimental data from the urban vertical farm of the Federal Research Center “Fundamentals of Biotechnology” of the Russian Academy of Sciences and literature data on the dynamics of the specific rate of CO_2_ absorption by potato leaves depicted in Figure 2a.

The final mass of tubers was chosen as the target parameter for verification, since it can be both calculated in accordance with the given model and easily measured in an experiment. To calculate in accordance with Formula (13), data from the literature [35] were used, reflecting the dynamics of the specific (per unit area of leaves) rate of CO_2_ absorption by potato leaves. To apply these data on the dynamics of the CO_2_ absorption rate, Equation (13) was converted to the integral form:

(14) $$m_{tubers}\left(t\right)=m_{tubers}\left(0\right)+\frac{6}{\eta_{starch}}S\cdot \overset{t}{\underset{0}{\int }}\frac{dm_{CO_{2}}\left(t^{\prime }\right)}{dt^{\prime }}dt^{\prime }$$

where S is the area of potato leaves, and $\frac{dm_{CO_{2}}\left(t^{\prime }\right)}{dt^{\prime }}$ is the dependence of the specific rate of CO_2_ absorption by potato leaves, the numerical values of which were obtained from [35] (see Figure 2a).

The calculation in accordance with the Formula (14) (Figure 2b) allowed to obtain the calculated prediction of the potato tuber mass grown in the vertical farm. The input parameters of the calculation were taken from the experiment: the initial mass of the planted tubers was $m_{tubers}\left(0\right)=225\;g$, the proportion of starch in the tuber η=0.2 [29], the average for the period of tuber formation (t=20 days [7])), and total leaf area $S=3800\;{\mathrm{cm}}^{2}$. The resulting final mass of tubers $m_{tubers}\left(t\right)$, calculated using these parameters, was 7.82 kg. The actual experimentally obtained mass of tubers was 5.24 kg, which is 1.49 times less than the calculated mass.

The difference between the calculated value and the experimental one of 1.49 times can be explained by the expenditure of CO_2_ for the formation of plant biomass different from tubers, such as the stem, leaves, and root system, together with the metabolism costs of CO_2_, that was not taken into account in the framework of the model under consideration. Thus, the data from [34] indicate that, when potatoes are grown at a temperature of 20–24 °C, which was also used in the considered experiments on growing potatoes in the urban vertical farm, the fraction of the total plant biomass is 1.46 times greater than the mass of tubers, which is close to the difference between the calculated mass and the experimental one within the framework of the model under consideration. Thus, in order to correctly determine the mass of tubers in the framework of the described model, it is necessary to take into account the expenditure of CO_2_ required for the formation of biomass different from tubers and CO_2_ consumption for plant metabolism.

## 4. Conclusions

Thus, the above analytical model of the relationship between the mass of tubers and the concentration of CO_2_ in a sealed room is correct and allows noninvasive determination of the potato tuber mass under the growing process with the use of measured dynamics of the CO_2_ concentration. Additional consideration of the CO_2_ expenditure for the formation of the remaining biomass of the plant makes it possible to clarify the mass of tubers calculated in the framework of the proposed model. Taking into account the CO_2_ exchange between the cultivation room and environment will allow the developed model to be expanded towards the case of growing in unsealed rooms. The described model opens opportunities for the implementation of contactless measurement of the potato tuber mass during the growing season when grown within various enclosed systems, such as closed vertical farms and greenhouses, as well as orbital and space growing systems. Moreover, the considered approach, based on a quantitative description of the synthesis of target component molecules from the carbon dioxide molecules during photosynthesis, can be extended to other cultivated plants, which will allow for a given chain of chemical reactions of the target component synthesis to obtain the analytical dependence of its mass on the concentration of CO_2_ in the room, as was done in this work for starch.

## Data Availability

The data available upon reasonable request.
